# Colossal magnetic fields in high refractive index materials at microwave frequencies

**DOI:** 10.1038/s41598-021-01644-1

**Published:** 2021-12-06

**Authors:** B. Luk`yanchuk, L. M. Vasilyak, V. Ya. Pecherkin, S. P. Vetchinin, V. E. Fortov, Z. B. Wang, R. Paniagua-Domínguez, A. A. Fedyanin

**Affiliations:** 1 Faculty of Physics, Lomonosov Moscow State University, Moscow, Russia 119991; 2 Joint Institute for High Temperatures, Russian Academy of Sciences, Moscow, Russia 125412; 3 School of Computer Science and Electronic Engineering, Bangor University, Bangor, LL57 1UT Gwynedd UK; 4 Institute of Materials Research and Engineering, A*STAR (Agency for Science, Technology and Research), Singapore, 138634 Singapore

**Keywords:** Micro-optics, Micro-optics

## Abstract

Resonant scattering of electromagnetic waves is a widely studied phenomenon with a vast range of applications that span completely different fields, from astronomy or meteorology to spectroscopy and optical circuitry. Despite being subject of intensive research for many decades, new fundamental aspects are still being uncovered, in connection with emerging areas, such as metamaterials and metasurfaces or quantum and topological optics, to mention some. In this work, we demonstrate yet one more novel phenomenon arising in the scattered near field of medium sized objects comprising high refractive index materials, which allows the generation of colossal local magnetic fields. In particular, we show that GHz radiation illuminating a high refractive index ceramic sphere creates instant magnetic near-fields comparable to those in neutron stars, opening up a new paradigm for creation of giant magnetic fields on the millimeter's scale.

## Introduction

**Figure 1:**
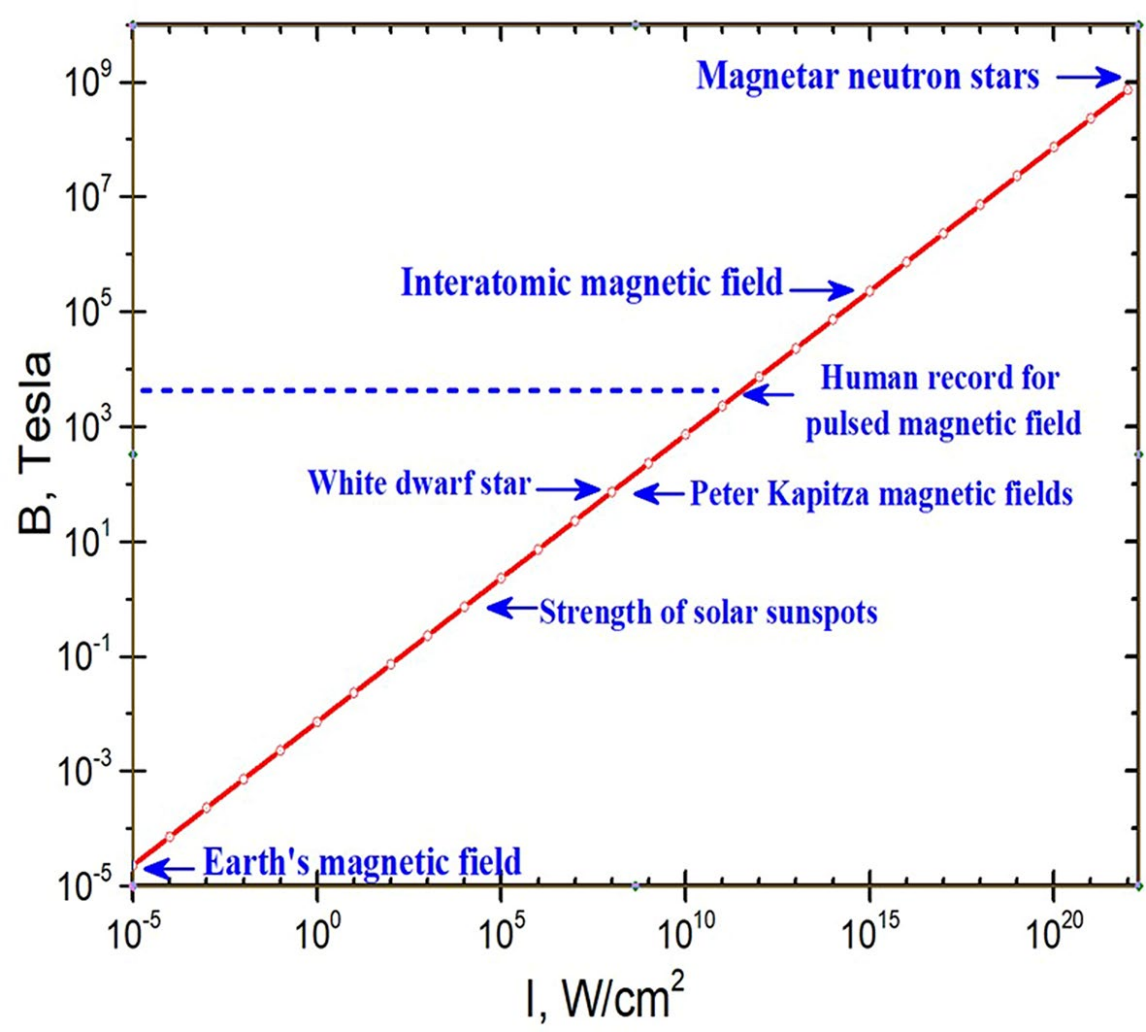
Schematic for the amplitude of magnetic induction versus light intensity.

Among the diverse forms of energy, a special place is given to the magnetic energy of an electromagnetic wave, associated with the strength of the magnetic field *H*. As it is known, a magnetic field arises as a result of a time-varying electric field (for example, in an electromagnetic wave) or it is generated by the movement of electric charges (for example, by a current in a solenoid). The idea of the energy of the light field follows from the Maxwell equations, according to which the volumetric energy density of an electromagnetic wave for non-dispersive, non-absorbing media is the sum of the energies of the electric, $\varepsilon \varepsilon_{0} E^{2} /2$, and magnetic, $\mu \mu_{0} H^{2} /2$, fields. In a vacuum, these two energies are equal to each other and, accordingly, 50% of the light intensity, $I$ , in a plane electromagnetic wave is presented in the form of magnetic energy. Therefore, high-frequency fields with large amplitudes of the magnetic field strength can be realized in intense light beams (from a physical point of view, it is better to speak not about the magnetic field $H$, but about the magnetic induction, $B = \mu \mu_{0} H).$ Accordingly, $B = \sqrt {{{2\mu_{0} I} \mathord{\left/ {\vphantom {{2\mu_{0} I} c}} \right. \kern-\nulldelimiterspace} c}}$, where *c* is the speed of light. At present, the record intensities achieved at the focus of the laser beam are *I* ~ 10^21^ ÷ 10^22^ W/cm^2^. When a substance is heated by such pulses, temperatures of tens and hundreds of millions of degrees are reached, which corresponds to the temperatures of initiation of nuclear reactions. Therefore, one of the most important promising applications of ultra-high-power petawatt lasers is laser controlled thermonuclear fusion^1^. The amplitude of the magnetic induction which can be reached in such beams is over 10^9^ Tesla (see Fig. 1). Note that the magnetic fields in magnetar neutron stars reach 10^11^ Tesla^2^, and the Planck limit for magnetic induction is 10^53^ Tesla^3^. For comparison, the Earth's magnetic field consists of an average strength of about 30 μT, which is also the typical magnitude of magnetic fields in the interstellar medium^4^. Astronomical objects such as white dwarfs consist of about 100 T.

There is a long staying problem of generation of high magnetic fields with field strength above 3000 T which presents a human record for pulsed magnetic fields^5,6^. To obtain super strong magnetic fields under laboratory conditions is a difficult task (the interested reader can find a historical perspective in, e.g., in Ref.^7^). The strongest continuous magnetic fields, of 45 T, have been produced using a Bitter magnet inside a superconducting magnet^7^. Magnetic fields above this level were created just in pulsed modes when strong electric currents were transmitted through solenoids of various designs, or when a magnetic flux inside a closed conducting coil (liner) was compressed by external forces. In this regard, the first system was created in 1924 by Peter Kapitsa^8^, who measured magnetic fields up to 10^6^ Gauss (100 T)^9^. In 1951, Andrei Sakharov suggested the idea of magnetoimplosive generators, which allows to obtain fields around 3000 T^5,6^. It should be noted that there are some physical limitations as to the maximal magnetic fields which can be reached using this principle, mainly related to the dissipation associated with the current flowing in the metals^8,9^, as well as with instabilities arising during the compression of the liner, caused by magnetic pressure effects^5,6^.

New physical principles are necessary to generate magnetic fields above 10^4^ T. In this sense, one possibility is, e.g., using magnetic field generation in laser-driven, colliding ultra-relativistic plasma pairs^10^. At extremely high laser wave intensities, the absorption of radiation can be effectively provided by radiation friction, instead of electron collisions. This permits generation of a super-strong magnetic field on the basis of inverse Faraday effect, with field strengths on the level of 10^5^ T at laser intensities above 10^23^ W/cm^2^ ^11^. For comparison, the amplitude of the magnetic component of light in a plane wave with such intensities exceeds 10^9^ T, and the electrical component of light reaches 3 × 10^12^ V/cm, which is steel three orders of magnitude lower than the vacuum breakdown threshold (10^16^ V/cm).

Returning to the fundamentals, the amplitude of the magnetic field at the center of a circular current loop can be written, on the basis of the Bio-Savart formula given by $B = \mu_{0} I/2R$, where *B* is the magnetic flux density in the center of the loop (measured in tesla in SI units), *I* is the electric current, *R* is the radius of the loop and *μ*_0_ = 4*π* × 10^−7^ H/m is the magnetic permeability of vacuum. From this equation one can see that an increase in the amplitude of the magnetic field can be obtained either by increasing the current, or by reducing the size of the coil. Thus, under simple, focused laser irradiation, the minimum size of the equivalent coil will be of the order of the wavelength, $\lambda$. This limit, which is related to the diffraction limit and how tightly an electromagnetic wave can be focused, applies only to propagating waves and can, therefore, be broken if one includes in the picture the non-propagating, evanescent components of the wave. These components are present, e.g., in the near-field of any scattering object, are at the very core of the present work and have been previously exploited to concentrate electromagnetic energy beyond diffraction limit and to enhance the radiation intensity in a variety of situations.

Among the first fields in which evanescent components of electromagnetic waves played a pivotal role is plasmonics. In this regard, the scattering of light by plasmonic nanoparticles allows creating optical nanovortices around the singularities where the Poynting vector tends to zero^12–14^. These vortices have characteristic scales $R \ll \lambda$ and, therefore, open the possibility for high magnetic fields. One possible way to reach a high localization of the vortices is via Fano resonances^15,16^, for which increasing localization is possible by considering increasingly higher multipolar orders^17–19^. Examples of these are those quadrupolar, octupolar, hexadecapolar, and even triacontadipolar resonances that can be excited in optimized disk-ring silver plasmonic nanostructure^20^. In plasmonic systems, however, higher order resonances are limited by the large dissipation associated with metals, a problem that is particularly acute in the visible range.

One possibility to avoid large dissipative losses is replacing metals by transparent dielectrics. In this regard, it was first theoretically shown^21,22^ and then experimentally demonstrated^23,24^ that high refractive index particles of sub-wavelength sizes can present a strong magnetic response to an incident electromagnetic wave. The presence of the magnetic family of multipoles on top of the usual electric ones, and the ability to control their amplitudes and phases, yields a wealth of unique optical properties to this type of particles, including strong directionality effects^25–27^, creation of magnetic hotspots^28^, realization of nonradiating modes^29–33^, low loss transmission lines^34^ and generalized Brewster’s effects^35^, among others^36–38^. As a consequence, all-dielectric resonant nanostructures have emerged as a promising platform for a number of nanophotonics applications, see e.g.^39–41^.

Beyond particles of sub-wavelength sizes, the ability of a large transparent sphere (as compared to wavelength) to focus light has been known for thousands of years. In modern photonics, this effect has come under the name of *photonic nanojet*. Interestingly, it has been recently shown that the magnetic component of light in nanojets can be amplified more than the electric component, i.e., that a magnetic nanojet can be “brighter” than an electric one^42^.

Even larger magnetic fields can be created inside the dielectric sphere^43^, due to the possibility of creating small optical vortices stemming from superoscillation effects^44^, with large phase gradients in the vicinity of singularities leading to equally large local values of the wave vector^45,46^. However, in this situation, dissipation plays a key role, and even the smallest values can greatly suppress these effects. As an example, it was recently shown^43^ that magnetic super resonances excited in a large particle with refractive index $n = 4$, which in the absence of dissipation lead to magnetic field enhancements $H^{2} \simeq 10^{7}$, can be reduced by two orders of magnitude with dissipation levels as low as $\tan \delta \approx 10^{ - 5}$ and completely disappear for values $\tan \delta \approx 10^{ - 3}$. For comparison, the best electrical insulation materials in engineering have $\tan \delta \approx 10^{ - 5} - 10^{ - 4}$. In this regard, it is interesting to analyse the generation of high magnetic fields in GHz region, where there are a number of ceramic materials with high dielectric constant and low loss (see e.g.^47,48^). This is what we do in the following, and demonstratre experimentally the generation of ultra-strong magnetic fields in the near-field of particles made of these materials.

## Results

First, we analyze theoretically the behavior of a nonmagnetic,$\mu = 1$, non-dissipative, ${\text{Im}} \varepsilon = 0,$ ceramic spherical particle with permittivity $\varepsilon = 150$. This value of the dielectric constant in the microwave region is typical for a number of high-voltage ceramics, for example, standard titanate-based ceramics (TiO_2_, CaTiO_3_, SrTiO_3_), such as the ones used in our experiments^47^. According to the Mie theory^49^ the scattering efficiency of the sphere, when illuminated by a plane wave, can be written as $Q_{sca} = {{\sigma_{sca} } \mathord{\left/ {\vphantom {{\sigma_{sca} } {(\pi R^{2} )}}} \right. \kern-\nulldelimiterspace} {(\pi R^{2} )}}$ ($\sigma_{sca}$ is the scattering cross section, $\pi R^{2}$ is the geometrical cross section of the particle). It also can be expressed as a sum of electric, $Q_{\ell }^{\left( e \right)} ,$ and magnetic, $Q_{\ell }^{\left( m \right)}$, partial scattering efficiencies, associated with the multipolar moment of $\ell$ th order multipoles:

1 $$Q_{sca} = \sum\limits_{\ell = 1}^{\infty } {\left( {Q_{\ell }^{\left( e \right)} + Q_{\ell }^{\left( m \right)} } \right),\quad } Q_{\ell }^{\left( e \right)} = \frac{2}{{q_{m}^{2} }}\left( {2\ell + 1} \right)\left| {a_{\ell } } \right|^{2} ,\quad Q_{\ell }^{\left( m \right)} = \frac{2}{{q_{m}^{2} }}\left( {2\ell + 1} \right)\left| {b_{\ell } } \right|^{2} ,$$

where the electric $a_{\ell }$ and magnetic $b_{\ell }$ scattering amplitudes are defined by formulas

2 $$a_{\ell } = \frac{{\Re_{\ell }^{\left( a \right)} }}{{\Re_{\ell }^{\left( a \right)} + i\Im_{\ell }^{\left( a \right)} }},\quad b_{\ell } = \frac{{\Re_{\ell }^{\left( b \right)} }}{{\Re_{\ell }^{\left( b \right)} + i\Im_{\ell }^{\left( b \right)} }},$$

with $\Re_{\ell }^{{\left( {a,b} \right)}}$ and $\Im_{\ell }^{{\left( {a,b} \right)}}$ coefficients expressed by

3 $$\begin{aligned} & \Re_{\ell }^{\left( a \right)} = n_{p} \psi_{\ell } \left( {q_{p} } \right)\psi^{\prime}_{\ell } \left( {q_{m} } \right) - n_{m} \psi_{\ell } \left( {q_{m} } \right)\psi^{\prime}_{\ell } \left( {q_{p} } \right), \\ & \Im_{\ell }^{\left( a \right)} = n_{p} \psi_{\ell } \left( {q_{p} } \right)\chi^{\prime}_{\ell } \left( {q_{m} } \right) - n_{m} \chi_{\ell } \left( {q_{m} } \right)\psi^{\prime}_{\ell } \left( {q_{p} } \right), \\ \end{aligned}$$

4 $$\begin{aligned} & \Re_{\ell }^{\left( b \right)} = n_{p} \psi_{\ell } \left( {q_{m} } \right)\psi^{\prime}_{\ell } \left( {q_{p} } \right) - n_{m} \psi_{\ell } \left( {q_{p} } \right)\psi^{\prime}_{\ell } \left( {q_{m} } \right), \\ & \Im_{\ell }^{\left( b \right)} = n_{p} \chi_{\ell } \left( {q_{m} } \right)\psi^{\prime}_{\ell } \left( {q_{p} } \right) - n_{m} \chi^{\prime}_{\ell } \left( {q_{m} } \right)\psi_{\ell } \left( {q_{p} } \right). \\ \end{aligned}$$

Here the functions $\psi_{\ell } \left( z \right) = \sqrt {\frac{\pi z}{2}} J_{{\ell + \frac{1}{2}}} \left( z \right)$ and $\chi_{\ell } \left( z \right) = \sqrt {\frac{\pi z}{2}} N_{{\ell + \frac{1}{2}}} \left( z \right)$ are expressed through the Bessel and Neumann functions^49^. We use the subscripts *m* and *p* to denote the values referring to the external media and the particle, with refractive indices $n_{m}$ and $n_{p}$, respectively. In the expressions above, $q_{m} = q\,n_{m}$ and $q_{p} = q\,n_{p}$. The symbol $q$ represents the so-called size parameter, defined as $q = {{\omega R} \mathord{\left/ {\vphantom {{\omega R} c}} \right. \kern-\nulldelimiterspace} c} = {{2\pi R} \mathord{\left/ {\vphantom {{2\pi R} \lambda }} \right. \kern-\nulldelimiterspace} \lambda }$. The power of Mie theory is that it allows identifying the spectral position, as well as the (electric or magnetic) multipolar origin of all resonances and the directional scattering effects^25^-^27^ (see Fig. S1 in Supplementary) observed in the scattering.

Beyond the far-field scattering characteristics, it is interesting to look at the electric and magnetic fields inside the particle. These, within the context of Mie theory, can be also computed through closed expression for the so-called, internal scattering amplitudes $c_{\ell }$ and $d_{\ell }$, given by^49^:

5 $$c_{\ell } = \frac{{in_{p} }}{{\Re_{\ell }^{\left( a \right)} + i\Im_{\ell }^{\left( a \right)} }},\quad d_{\ell } = \frac{{in_{p} }}{{\Re_{\ell }^{\left( b \right)} + i\Im_{\ell }^{\left( b \right)} }}.$$

Although the denominators of these amplitudes are the same as in amplitudes $a_{\ell }$ and $b_{\ell }$ in (2), which means that the position of these resonances are close, the numerators of (5) never tends to zero. As a result, the values of amplitudes $\left| {c_{\ell } } \right|^{2}$ and $\left| {d_{\ell } } \right|^{2}$ are not restricted to unity as $\left| {a_{\ell } } \right|^{2}$ and $\left| {b_{\ell } } \right|^{2}$, but increase with values of size parameter and refractive index. To compare both type of resonances it is convenient to introduce the partial internal scattering efficiencies, in a way similar to those in Eq. (1):

6 $$F_{\ell }^{\left( e \right)} = \frac{2}{{q_{m}^{2} }}\left( {2\ell + 1} \right)\left| {c_{\ell } } \right|^{2} ,\quad F_{\ell }^{\left( m \right)} = \frac{2}{{q_{m}^{2} }}\left( {2\ell + 1} \right)\left| {d_{\ell } } \right|^{2} .$$

It should be noted that the maximal efficiencies $Q_{\ell }^{{\left( {e,m} \right)}}$ and $F_{\ell }^{{\left( {e,m} \right)}}$ have different behaviour versus the order of mode: while $\max Q_{\ell }^{{\left( {e,m} \right)}}$ monotonously decreases when increasing the $\ell$ number, $\max F_{\ell }^{{\left( {e,m} \right)}}$ is extremely fast growing with $\ell$^43^.

**Figure 2:**
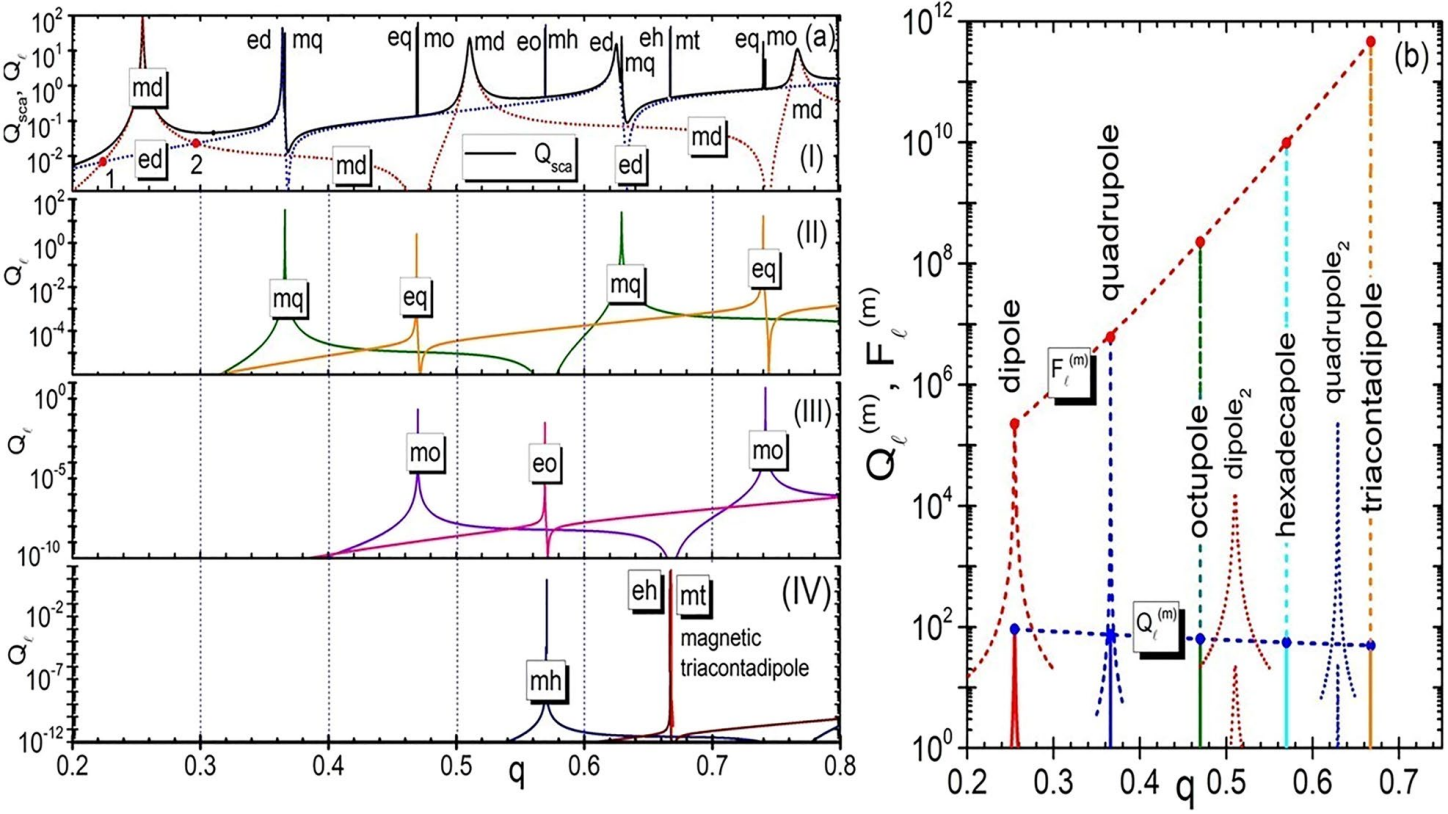
(**a**) The upper panel (I) shows the scattering efficiency (black line) together with electric dipole (ed), $Q_{1}^{(e)}$, (blue dotted curve) and magnetic dipole (md), $Q_{1}^{\left( m \right)}$, (red dotted curve) scattering efficiencies. the point “1” corresponds to “first Kerker condition”, when the back scattering is completely suppressed. The point “2” corresponds to the “second Kerker condition” with minimal forward scattering^25,26^. Panel (II) shows the partial scattering contributions of the electric (eq), $Q_{2}^{\left( e \right)}$, (orange), and magnetic (mq), $Q_{2}^{\left( m \right)}$, (olive), quadrupole terms. Panel (III), correspondingly, illustrates the contribution of electric (eo) and magnetic (mo) octupoles, $Q_{3}^{{\left( {e,m} \right)}} .$ Panel (IV) shows contribution of electric (eh), $Q_{4}^{\left( e \right)}$, and magnetic (mh), $Q_{4}^{\left( m \right)}$, hexadecapole terms. Additionally, contribution of magnetic triacontadipole (mt), $Q_{5}^{\left( m \right)}$ is also shown in this panel. All calculations are done for $\varepsilon = 150$. (**b**) Variation of maximal efficiencies $Q_{\ell }^{\left( m \right)}$ and $F_{\ell }^{\left( m \right)}$ for the for the first five modes $\ell \;\; = \;\;1,\;\;2,\,\;3,\,\;4,\,\;5$. The dotted curve (proportional to modulus of the amplitudes $b_{\ell }$ ) connects the maximal $Q_{\ell }^{\left( m \right)}$ values changing from $Q_{1}^{\left( m \right)} =$ 92.3 to $Q_{5}^{\left( m \right)} =$ 49.3. The dotted red curve (proportional to modulus of the amplitudes) connets the maximal $F_{\ell }^{\left( m \right)}$ values changing from $F_{1}^{\left( m \right)} = 2.24 \cdot 10^{5}$ to $F_{5}^{\left( m \right)} = 4.3 \cdot 10^{11}$.

In Fig. 2a, we plot the total scattering efficiency and the different partial contributions for a sphere with $q \le 0.8$. In the plot, all the different resonances observed are identified and labelled correspondingly. One can see two interesting phenomena when looking at this plot. The first, is the periodic repetitions of the observed resonances, which start from the lowest-order, magnetic dipole one. The second is some kind of interplay between resonances, that tend to bunch together^50^. See, e.g., the electric dipole (ed) resonance, which is excited always in close proximity to the magnetic quadrupole (mq) one. The same happens for the electric quadrupole (eq) and the magnetic octupole (mo), the electric octupole (eo) and the magnetic hexadecapole (mh), etc. As expected, the quality (Q) factor of the resonances increases with their order, i.e., the linewidth of high order resonances become increasingly narrow, a situation that happens both for plasmonic^15^ and high refractive index dielectric particles^20^. In Fig. 2b, we show a comparison between the evolution of the partial scattering efficiencies associated to the magnetic multipoles $Q_{\ell }^{\left( m \right)}$ and the evolution of the corresponding partial internal scattering efficiencies $F_{\ell }^{\left( m \right)}$. As can be seen, while the scattering efficiencies do not significantly change with the increase in order, there is a very pronounced effect in $F_{\ell }^{\left( m \right)}$ increasing up to ten orders of magnitude when $\ell$ varies from 1 to 5. From Fig. 2b follows that the fields inside the particle can be much higher than in the far field region. A comparison between the electric and magnetic field distributions inside the particle for the magnetic dipole and magnetic quadrupole modes is shown in Supplementary Figs. S2 and S3.

**Figure 3:**
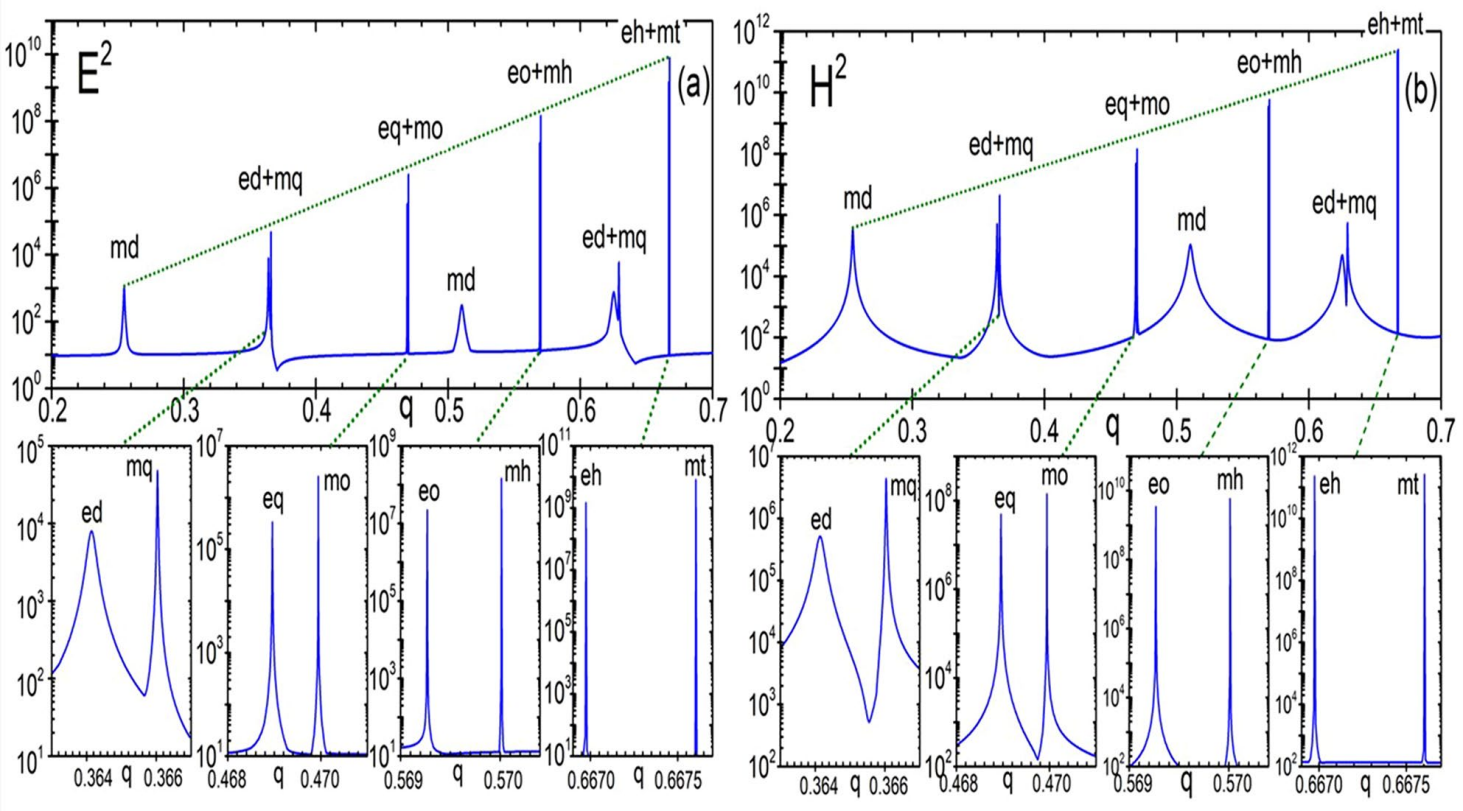
Maximal intensity E^2^ (**a**) and magnetic intensity H^2^ (**b**) growth from magnetic dipole to magnetic triakontadipole resonances.

**Figure 4:**
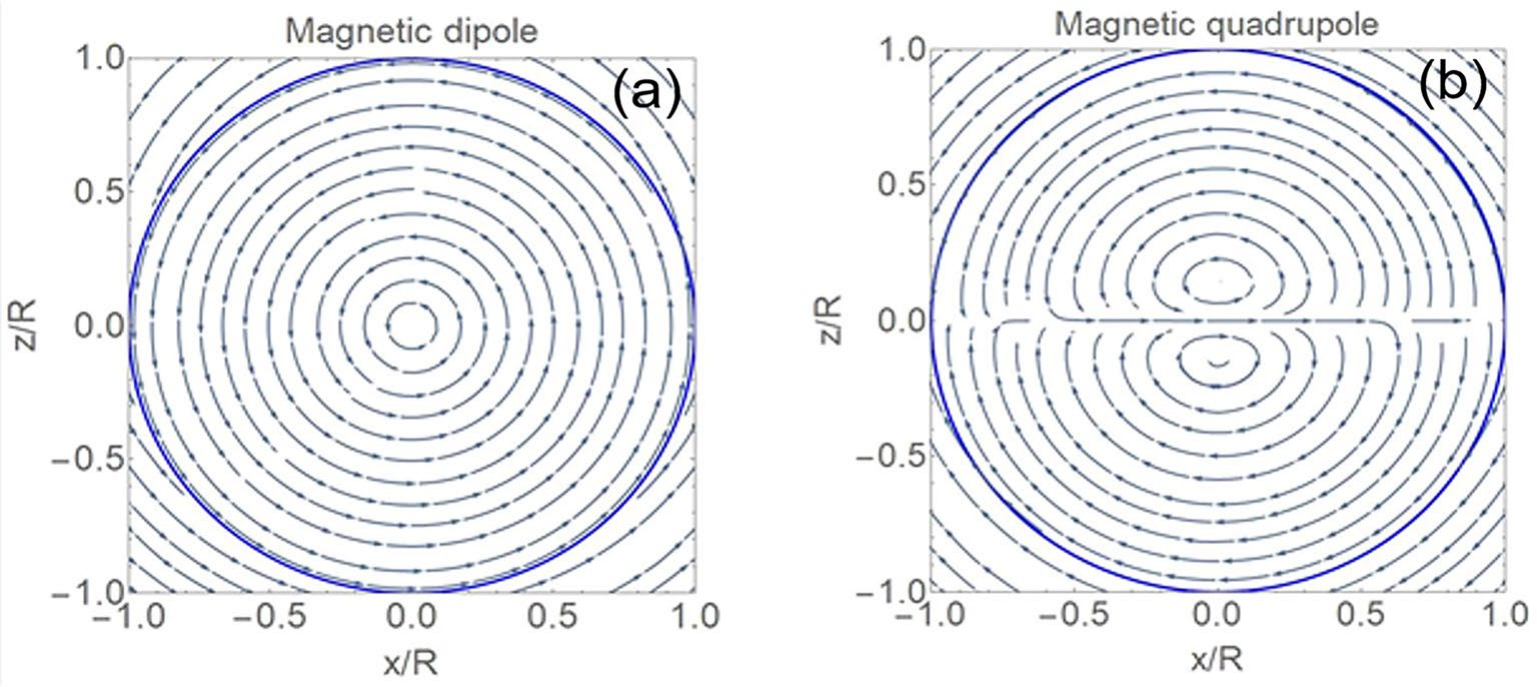
Distribution of the electric field streamlines within the *xz* plane at the magnetic dipole resonance, *q* = 0.254905 (**a**), and magnetic quadrupole resonance, *q* = 0.36604 (**b**). The plane wave is incident along the z-axis with its electric field polarized along the x-axis. Corresponding electric intensity distributions are shown in Figs. S2 and S3.

**Figure 5:**
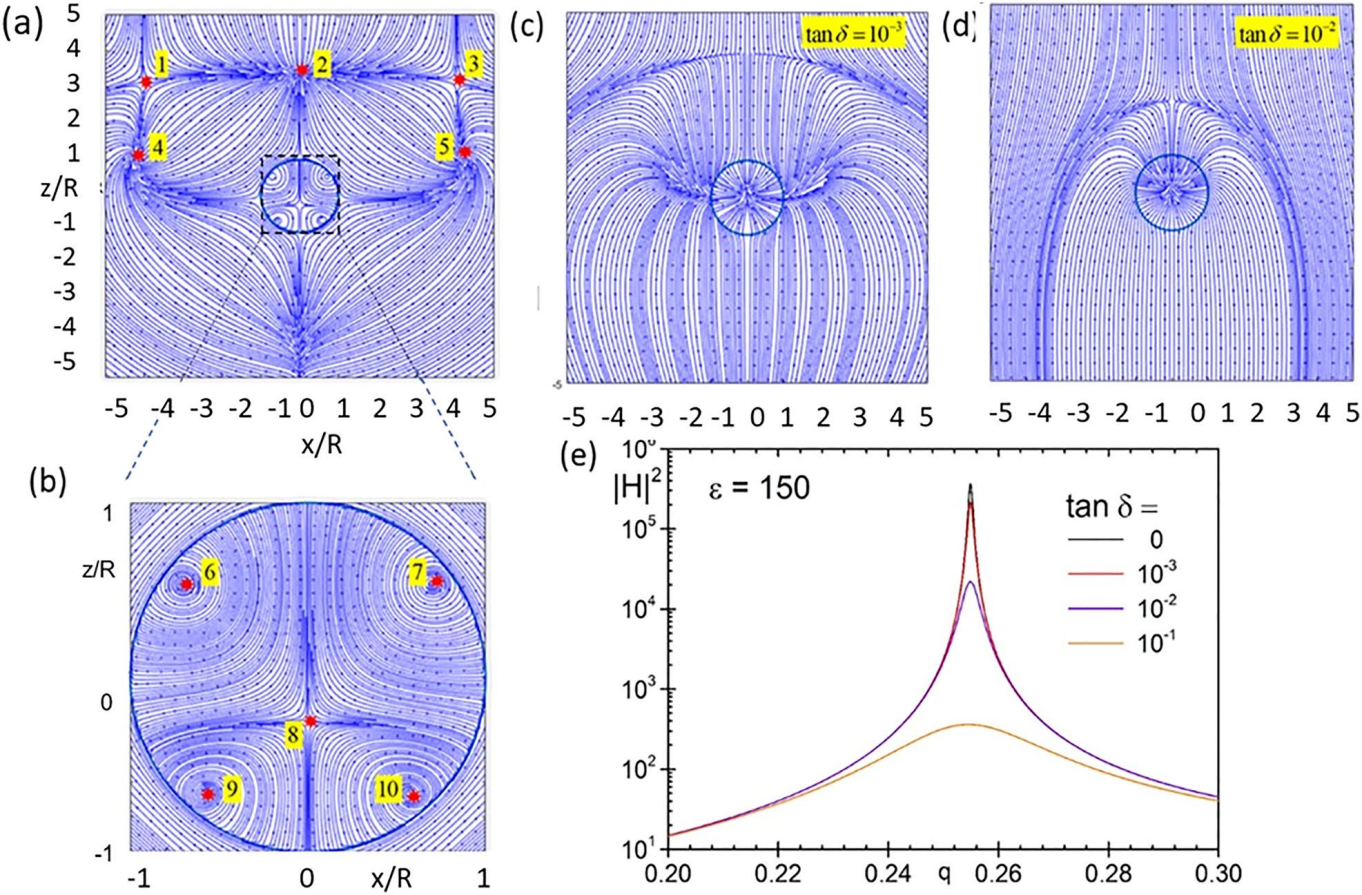
(**a**) and (**b**) Distribution of the Poynting vector field within the *xz* plane at the magnetic dipole resonance, *q* = 0.254905. Red dots indicate five singular points around the particle (left picture) and five singular points inside the particle. There are saddles (points 1,3,8) and nodes (2) and focal points (4,5,6,7,9,10). (**c**) and (**d**) Effect of dissipation for the case of a magnetic dipole resonance $q = 0.2549$ excited in a particle with $\varepsilon = \varepsilon^{\prime} + i\varepsilon^{\prime\prime}$, $\varepsilon^{\prime} = 150$ and different values of loss tangent $\tan \delta = \varepsilon^{\prime\prime}/\varepsilon^{\prime}.$ Dissipation with $\tan \delta = 10^{ - 3}$ does not influences maximal magnetic field practically. However, $\tan \delta = 10^{ - 2}$ yields about two order of magnitude suppression in the maximal field values (**e**).

The maximum intensities of the electric and magnetic fields are plotted in Fig. 3 as a function of the size parameter, corroborating the expected rapid increase of the intensity with the mode number. It can be seen from the figure that the magnetic intensity enhancement for the first magnetic dipole resonance exceeds 10^5^ and further increases by almost an order of magnitude with each subsequent multipole resonance. High resonances become very narrow. As already mentioned, the reason for the formation of high magnetic fields in dielectrics is associated with the formation of highly localized optical vortices in the material. These vortices can be seen from the electric field streams in Fig. 4, as well as in the distribution of the Poynting vector, both around and inside the particle, as seen in Fig. 5.

The main limiting factor when trying to generate high magnetic fields are dissipation effects. As one can see in Fig. 5, even if low dissipation levels ($\tan \delta = 10^{ - 3}$) do not influence the maximal magnetic fields associated to the magnetic dipole resonance, it can strongly affect the values obtained with higher order resonances, since they are more sensitive to dissipation^43^.

Fortunately, there are many materials with high dielectric constant and low loss in the GHz spectral range^51,52^. In this regard, some ceramics in that range might even have what is usually called a "colossal" dielectric constant, reaching extremely high values of $\varepsilon > 10^{4}$^53^. In our experiments, we use more standard, titanate-based ceramics (TiO2, CaTiO3, SrTiO3). These are commonly used for the production of high-voltage capacitors K15U-2, with dielectric constant varying in the range $\varepsilon = 130 - 180$ and with an associated loss tangent in the range $\tan \delta = 10^{ - 4} - 10^{ - 3}$ ^51^. For the sample used in our experiments, we measured a loss tangent of our ceramics was only $\tan \delta = 3 \times 10^{ - 4}$ at a frequency of 1 kHz. According to Ref.^51^, this loss tangent practically does not change up to a frequency of 1 GHz and increases significantly only at a frequency of 10 GHz.

## Methods

Studies of the magnetic field distribution can be performed on the basis of spectroscopic data, which allows defining both ${\text{Re}} \varepsilon$ and $\tan \delta$ and, in turn, finding the electric and magnetic fields. This technique is used in astronomy for measuring magnetic fields in stars and galaxies^2,4^. If one is interested in measuring the near-fields, a local probe is necessary. In the case of nanostructures, this probe is typically the tip of a near field scanning optical microscope (NSOM). Metal-coated, aperture-type NSOM tips are particularly sensitive to the magnetic fields and have indeed been used to measure hotspots in the near magnetic field of silicon dimers at visible wavelengths^28^. In the case of GHz waves, the millimeter—centimeter scale structures with high dielectric constant allows measuring the spectrum of scattering using a local magnetic field sensor^54–56^. In our case, this sensor takes the form of a small metallic loop, which can efficiently measure the magnetic field component parallel to its axis (i.e., perpendicular to the loop plane). When the plane of the sensor loop is placed near the sphere and oriented perpendicular to the incident wave vector **k** (i.e., parallel to the plane containing the incident **H** and **E** fields), we do not measure the magnetic field **H** of the incident wave. Since the scattered fields may have all different directions, using this configuration its value at this position can be registered, while the signal from a plane EM wave in this position is greatly weakened. In our experiments (see Supplementary Information for details), the spectrum analyzer measures the power attenuation coefficient $a$ in decibels (dB) unit. Coefficient $a$ is defined by the expression $a = 10\;\log \,\,\left( {{{P_{1} } \mathord{\left/ {\vphantom {{P_{1} } {P_{0} }}} \right. \kern-\nulldelimiterspace} {P_{0} }}} \right)$, where $P_{0}$ and $P_{1}$ are the measured signal powers without ceramic sphere and with ceramic sphere, respectively.

**Figure 6:**
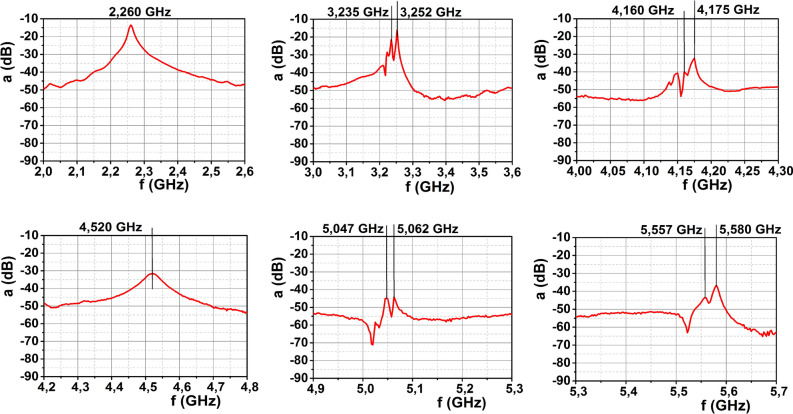
Experimentally measured GHz spectra in the vicinity of reference frequency values.

Using this technique, we measured the scattering spectrum of a ceramic sphere with a diameter 10.95 mm. Figure 6 shows the results in the vicinity of reference frequency values used to calibrate the measurement of permittivity. As seen from the plots, we were able to see all resonances depicted in Fig. 2, which gave us the ability to precisely specify the dielectric function of the ceramic material. This is done through a simple fitting to Mie theory predictions. Since we know the size of the particle, a diameter 10.95 mm, and the experimentally measured resonant frequencies, we can find a suitable dielectric function by just a fitting, whose results yields the value $\varepsilon = 144.68$ for the dielectric constant. Moreover, the experiment shows equidistantly-spaced resonant frequencies for the magnetic and electrical vibrations for various modes, in good correspondence with the repeated electric and magnetic dipole resonances predicted by theory. Incidentally, we also confirmed experimentally the scattering directivity effect associated with the realization of the so-called Kerker’s conditions, as seen in Supplementary Fig. S1. At 2.2 GHz one can see almost zero back scattering due to first Kerker condition while, at 2.36 GHz the realization of the second Kerker condition is met, translated in this realization as minima in the forward scattering.

**Figure 7:**
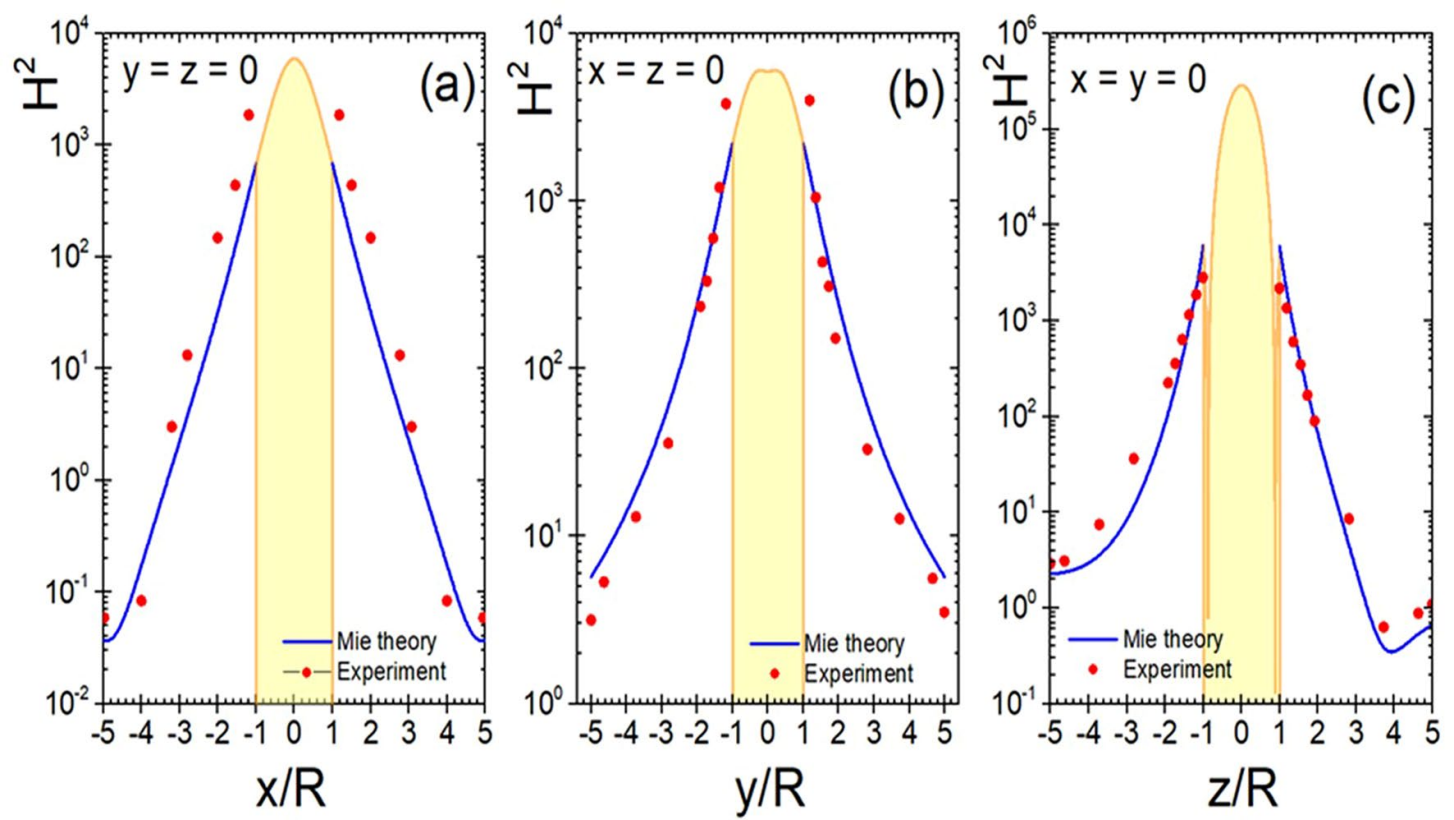
Magnetic intensity distribution within the particle with $\varepsilon = 144.68$ at the frequency 2.26 GHz (magnetic dipole resonance) along the *x*-axis at *y* = *z* = 0 (**a**), along the *y*-axis at *x* = *z* = 0 (**b**), and along the *z*-axis at *x* = *y* = 0 (**c**). Blue solid lines are distributions of magnetic intensity around the particle according the Mie theory. Red dots are the experimental points measured by magnetic probe sensor. In the center of the particle maximum magnetic enhancement according to the Mie theory reaches *H*_2_ = 3.25 × 10^5^.

The collected spectroscopic data provides evidence of a huge enhancement of the magnetic intensity H^2^ of about 5 orders of magnitude for the magnetic dipole resonance at 2.26 GHz and up to 8 orders of magnitude for the electric quadrupole resonance near 4.2 GHz. These values,​which ​correlate well with previously predicted results^43^, are achieved in a situation in which the length of the exciting plane EM wave is larger than the element size and in which the size of the electric and magnetic field probes is much smaller. Such an experimental setup allows not only measuring resonant frequencies, but also measuring the EM field distributions, as well as exploring the interaction of several elements. In particular, using sufficiently small probes and drilling a narrow channel in the sphere, it could be possible even to “travel to its center” as to measure the internal fields of the particle. This fields might get even slightly enhanced in this situation as compared to those of the solid sphere^57^ (see Supplementary Fig. S5 for the theoretical prediction when a small channel of 150 μm diameter drilled in the ceramic particles is considered). According to Mie theory, we can see strong magnetic fields in the vicinity of the particle surface. In Fig. 7, we show that this is indeed the case by plotting the magnetic intensity distribution around the ceramic particle with diameter 10.95 mm and $\varepsilon = 144.68$ at a frequency of 2.26 GHz (corresponding to the magnetic dipole resonance). In this plot, the yellow region indicates the region inside the particle, the blue solid lines show the magnetic intensity computed using Mie theory and the red dots present the experimental points measured by our magnetic probe sensor. As can be seen, the agreement is remarkable; the small deviation from the Mie theory being related to the finite probe size. These data yields clear indication that, even for the lowest order, magnetic dipole resonance, extremely large enhancement values of up to four orders of magnetic intensity can be achieved. It is easy to estimate from the Mie theory the maximum magnetic intensity inside the particle at the magnetic octupole resonance (this resonance is well recognized in experimental data Fig. 6). It consists field enhancement of above $H^{2} = \;5 \times 10^{7}$. In fact further increase in magnetic field depends on a dissipation parameter $\tan \delta$—how small it can be for higher order resonances at GHz frequency.

## Conclusion

To summarize, we have confirmed experimentally and numerically the emergence of huge magnetic field enhancements within and around high refractive index particles. This is achieved in connection with the creation of optical vortices in their near-field when high-order resonances are excited in these particles. While prone to disappear when even mild dissipation is present, which might make difficult its observation at optical frequencies, these huge magnetic field enhancements are readily available in the GHz range, where one could even potentially benefit from those material systems exhibiting “colossal” permittivities. In this manuscript we verified experimentally a key point relate to magnetic field enhancement 10^5^ compared to magnetic field in plane electromagnetic wave. Theoretically the incident magnetic field can be enhanced by 10^6^–10^10^ times inside the microwave materials with $\varepsilon \approx 140 - 150$. There are many possible applications for magnetic nanojets, including enhancement of radiation emission from magnetic emitters, magnetic absorption, and magnetic nonlinear optical effects. Other possible applications are: spectroscopy, diagnostics based on the Zeeman effect, electron paramagnetic resonance, investigation of the effect of strong magnetic fields on materials (semiconductors) and microchips; devices based on superconductors in a magnetic field, for example, creating current interrupters in a thin superconducting filament.

## Supplementary Information

Supplementary Information.
