# Supplementary material for: Colossal magnetic fields in high refractive index materials at microwave frequencies
Source: Sci Rep. 2021 Dec 6;11:23453. doi: 10.1038/s41598-021-01644-1 (PMC8648870; doi:10.1038/s41598-021-01644-1)
Supplement: Supplementary file 1 — Supplementary Information. [file 41598_2021_1644_MOESM1_ESM.pdf]

B. Luk`yanchuk[1*], L.M. Vasilyak[2], V.Ya. Pecherkin[2], S.P. Vetchinin[2], V.E. Fortov [2], Z.B. Wang[3], R. Paniagua-Domínguez[4], A.A. Fedyanin[1]

[1]Faculty of Physics, Lomonosov Moscow State University, Moscow, 119991, Russia

[2]Joint Institute for High Temperatures, Russian Academy of Sciences,125412 Moscow, Russia

[3]School of Electronic Engineering, Bangor University, Bangor, Gwynedd, LL57 1UT, UK

[4]Institute of Materials Research and Engineering, A*STAR (Agency for Science, Technology and Research), 138634 Singapore

Supplementary

(a) Scattering directivity

The forward scattering efficiency Q_{FS} as well as the backward, Q_{BS}, scattering efficiency in the Mie theory are represented by [1]

$$Q_{FS} = \frac{1}{q^2}\left|\sum_{\ell=1}^{\infty}(2\ell+1)(a_\ell + b_\ell)\right|^2, \quad Q_{BS} = \frac{1}{q^2}\left|\sum_{\ell=1}^{\infty}(2\ell+1)(-1)^\ell(a_\ell - b_\ell)\right|^2. \tag{S1}$$

For small particle with $q \ll 1$ the basic contribution in sums (S1) yields the dipole term with $\ell = 1$. For this case two directional resonances arise at $\mathrm{Re}\, a_1 = \mathrm{Re}\, b_1$. At $\varepsilon = 150$ this condition is fulfilled for $q = q_{K1} \approx 0.2240$ and $q = q_{K2} \approx 0.2965$, see the points "1" and "2" in panel (I) of Fig. 1. In Fig. S1(a) we show ratio of forward to back scattering $F/B \equiv Q_{FS}/Q_{BS}$ and inverse value $F \equiv Q_{BS}/Q_{FS}$ together with the corresponding polar scattering diagrams [2]. At $q = q_S \approx 0.25491$ one can see a symmetrical scattering; at $q < q_S$ the forward scattering dominates, at $q > q_S$ the back scattering dominates. At the case of forward scattering at $q = q_{K1}$ ratio Q_{FS}/Q_{BS} consist almost nine orders while at $q = q_{K2}$ ratio Q_{BS}/Q_{FS} consist just three order of magnitude. In general case, the scattered far-field from a dielectric sphere may vanish at any polar angle in either of the planes of oscillation of the induced dipoles, see the discussion in [3,4]. Directional scattering effect was confirmed experimentally, see in panel "e".

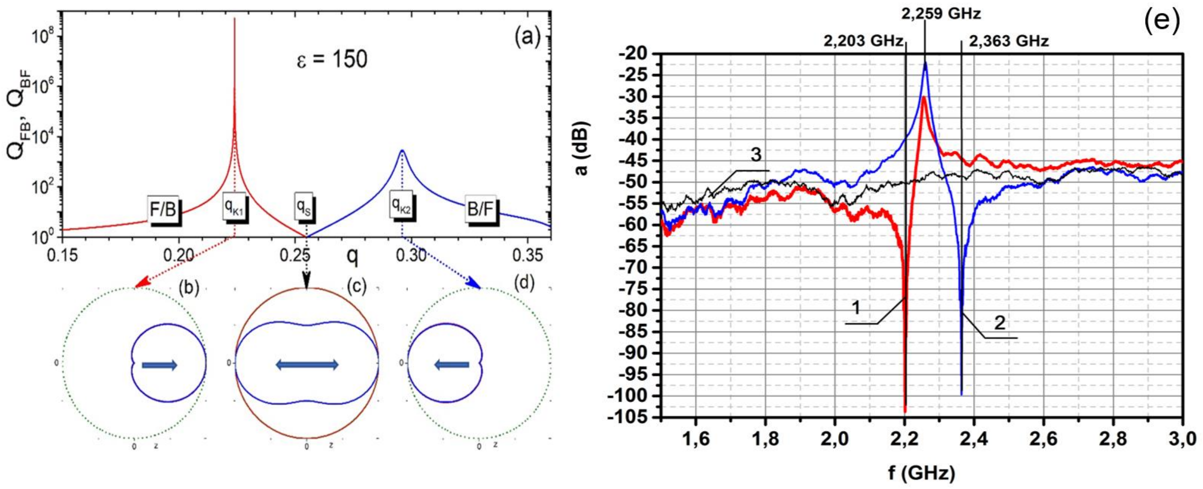

Fig. S1. (a) Directivity of scattering versus size parameter illustrated by ratio of Q_{FS}/Q_{BS} (red line) and ratio of Q_{BS}/Q_{FS} (blue line). (b), (c) and (d) are the polar scattering diagrams for $q = q_{K1}$ (forward scattering), for $q = q_S$ (symmetrical scattering) and for $q = q_{K2}$ (back scattering). Blue lines shows linearly polarized light; red lines represent non-polarized light. For directional scattering (panels (b) and (d)) scattering diagrams for polarized and non-polarized light are practically the same. Wave vector on scattering diagrams is directed from left to right. (e) Experimentally measured scattering intensity of dielectric sphere: 1 - the probe in front of sphere; 2 - the probe behind of sphere; 3 - without sphere.

(b) Field enhancement at magnetic dipole resonances

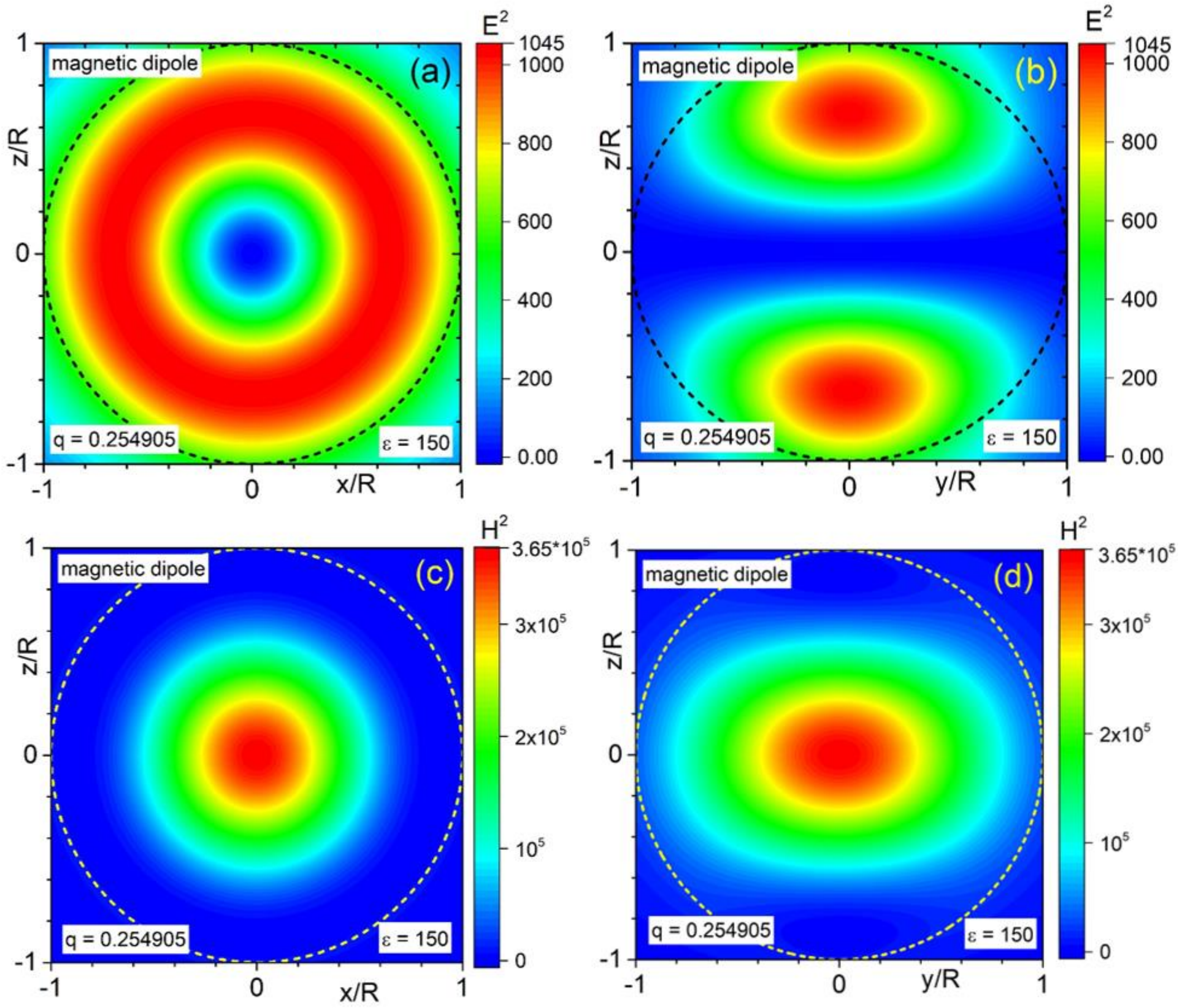

Fig. S2. Field enhancement distribution for a spherical particle with $\varepsilon = 150$ within the *xz* (a,c) and *yz* (b,d) -planes for electrical intensity E^2 (a,b) and magnetic intensity H^2 (c,d) for magnetic dipole resonance, $q \approx 0.2549$.

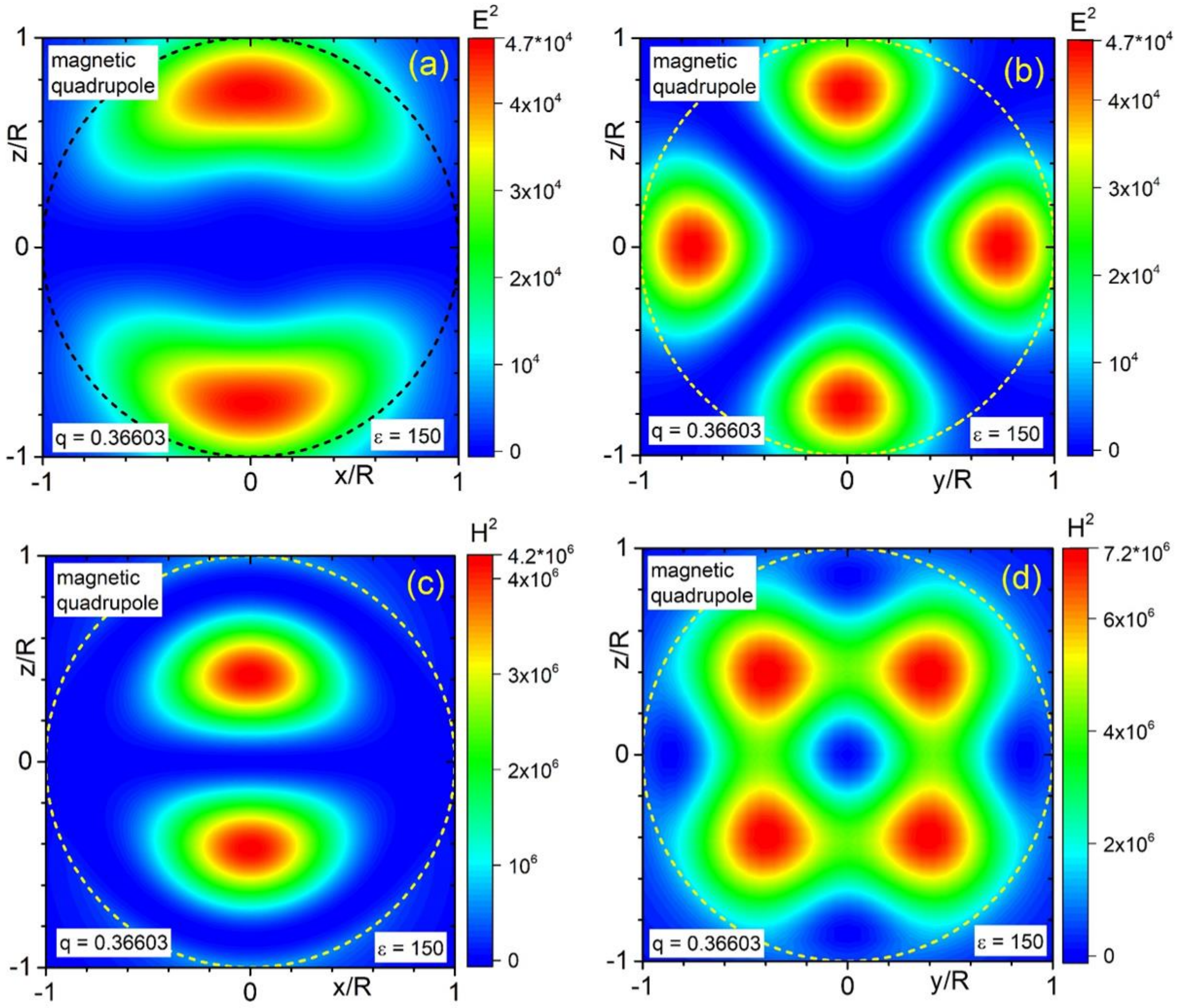

Fig. S3. Field enhancement distribution within the xz (a, c) and yz (b, d) -planes for electrical Intensity E^2 (a, b) and magnetic intensity H^2 (c, d) for magnetic quadrupole resonance, $q \approx 0.3660$.

(c) Gigahertz Resonant Measurement

The experimental setup used for the verification of the theoretical results is shown in Figure S4. The experiments were aimed at the excitation of resonant frequencies in the dielectric ceramic sphere irradiated by the linearly polarized microwaves. Agilent E5071C ENA Network Analyzer (1) was used for generation and registration of emission spectra of GHz-range. The transmitter power was 10 mw. ETS-Lindgren's model 3115 double ridged waveguide horn antenna was applied as an emitter (3). To increase the signal-to-noise ratio and decrease the influence of radio noise an additional 20 dB gain amplifier was used (2). The high frequency magnetic field in the near zone was registered by the screened loop magnetic field sensor Beehive Electronics 100B EMC Probe (4) with 3.7 mm loop diameter of sensitive element. The plane of the magnetic field sensor ring was directed along the magnetic field vector and electric field vectors of the incident microwave and perpendicular to the wave vector k.

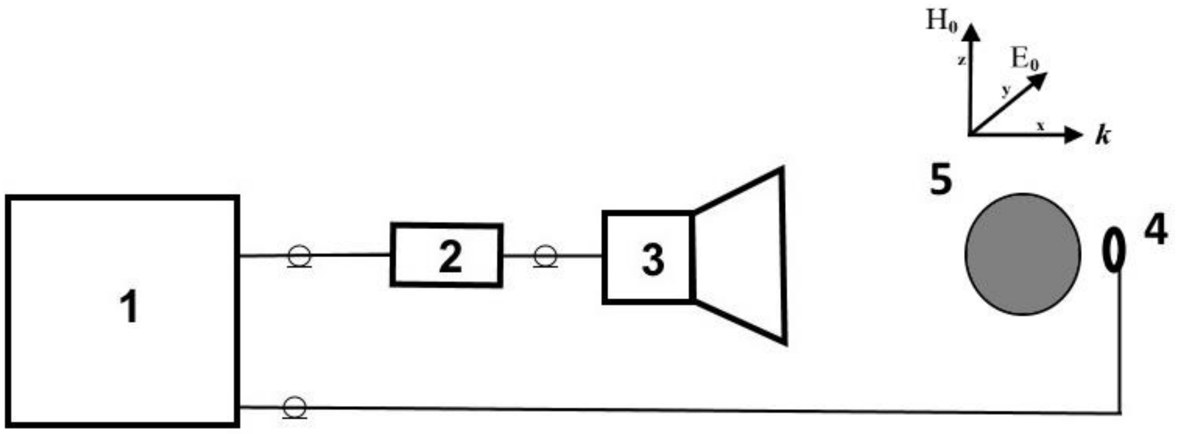

Fig. S4. Experimental setup. (1) Network analyzer. (2) RF broadband amplifier. (3) Double ridged waveguide horn antenna. (4) High frequency probe of magnetic field. (5) Dielectric sphere made of K15U-2 capacitor's ceramics.

The ceramic sphere with diameter of 10.95 ± 0.1 mm made of K15U-2 capacitor was used in these experiments. The value of the dielectric permittivity varied in the range ε = 140-170 with a loss tangent tan $\delta = 10^{-4}$-10^{-3}. The distance between the edge of antenna and the magnetic field sensor was 20 cm. The center of the dielectric sphere and the center of the magnetic field sensor loop were located along the wave vector emanating from the geometric center of the

antenna. The magnetic field probe was positioned 2 mm behind the ceramic sphere for the measured data in Fig. 5 (Main text) and the probe was positioned 2 mm in front of and behind the ceramic sphere for the data in Fig. S1.

For each test, the background radiation level was measured in the presence of incident microwave radiation without the ceramic sphere. The spectrum analyzer measures the power attenuation coefficient a in decibels (dB) unit. Coefficient a is defined by the expression $a = 10\lg(P_1/P_0)$, where P_0 and P_1 is the measured signal power without ceramic sphere and with ceramic sphere, respectively. Our setup permits to measure signal variation on the scale of 80 dB, see Fig. S1 (e).

(d) Influence of chanel through the particle on the magnetic field distribution

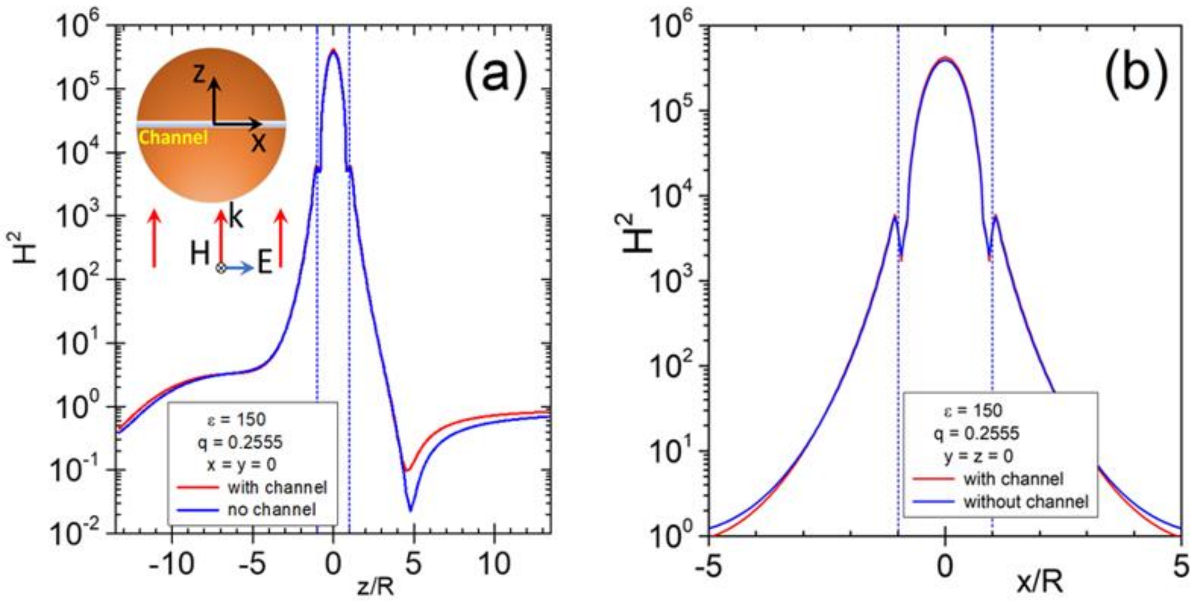

Fig. S5. Magnetic intensity distribution (a) along the *z*-axis at *x* = *y* = 0 within the particle with $\varepsilon = 150$ and size parameter $q = 0.2555$ at the dipole resonance. Insert shows the schematic for the sphere with channel which have been used in calculations. Distribution (b) along the x-axis at y = z = 0 within the particle. In the center of the solid particle maximum magnetic enhancement reaches $H^2 = 3.9 \times 10^5$. Adding a cylinder chanel one can increase this intensity up to $H^2 = 4.2 \times 10^5$. Calculations are performed with CST software. For the solid sphere it shows result close to the Mie theory (see in Fig. 4e).

References

[1] C. F. Bohren, D. R. Huffman, *Absorption and Scattering of Light by Small Particles* (Wiley–Interscience, New York, 2004).

[2] Y. H. Fu, A. I. Kuznetsov, A. E. Miroshnichenko, Y. F. Yu, B. Luk'yanchuk, *Directional visible light scattering by silicon nanoparticles,* Nat. Commun. **4**, 1527 (2013).

[3] R. Paniagua-Domínguez, B. Luk'yanchuk, A. Miroshnichenko, J.A. Sánchez-Gil, *Dielectric nanoresonators and metamaterials*, J. Appl. Phys. 126, 150401 (2019).

[4] R. Paniagua-Dominguez, B. Luk'yanchuk, A.I. Kuznetsov, *Control of scattering by isolated dielectric nanoantennas*. In Dielectric Metamaterials, pp. 73-108 (Woodhead Publishing, 2020).
